# 

**Published:** 1841-11

**Authors:** 


### CONTENTS

OF NO. V.

---

### ORIGINAL COMMUNICATIONS.

### ESSAYS AND CASES.

Art. I.—Observations on the epidemic Yellow Fever of Natchez, and of the South-west. By John W. Monette, M. D. &c., of Washington, Mississippi. - - 331

Art. II.—A Case of remarkable and speedy recovery from severe injury of the Spinal Column. By Solon Borland, M. D., of Memphis, Tenn. - - - - 356

Art. III.—Cases of Scrofula cured by the Xanthoxylum Fraxineum. - - - - - - - 361

Art. IV.—History of a Case of long standing Spinal Irritation, successfully treated. By John Dawson, Jr., of Jamestown, Ohio. - - - - - - 363

Art. V.—Ice in Cholera Infantum. By Dr. W. S. Lawrie, of New Concord, Ky. - - - - 366

### BIBLIOGRAPHICAL NOTICES.

Art. I.—A Treatise on the Cause of the Disease called by the People Milk Sickness; as it occurs in the Western and Southern States. By John Simpson Seaton, M. D., of Jefferson County, Ky. Louisville: pp. 49. 368

7

Art. II.—Physiology and Animal Mechanism. Philadelphia; 1841: pp. 101. - - - - - - 371
Art. III.—Bulletin of the Proceedings of the National Institution for the promotion of Science. Established at Washington in 1840: pp. 65. - - - 372

SELECTIONS FROM AMERICAN AND FOREIGN JOURNALS.

Effect of Belladonna upon an Irritable Eye. - - 373
Ergot. - - - - - - - - 374
Formulæ used in the Treatment of Tinea Capitis. - - 375
New Method of Applying Iodine for the Cure of Phthisis Pulmonalis. - - - - - - 375
Inhalation of Conium and Iodine in Tubercular Phthisis Pulmonalis. - - - - - - 375
Magendie's Method of treating Neuralgia. - - 378
On the Use of Ergot. - - - - - 380
Observations on Solar Asphyxia, Coup de Soleil or Sun Stroke. 384
Employment of Creosote for the cure of affections of the Eye. 394
Division of the Genio-hyo-glossi Muscles for Stammering. 395
Effects of Calculus in the Female Child. - - 396
Death by Irritation. - - - - - 398
Iodide of Potassium. - - - - - - 398

ORIGINAL INTELLIGENCE.

Publication of Medical Books in connexion with this Journal. 399
Progress of Science. - - - - - - 400
Sulphate of Quinine in Congestive Fever. - - 402
Urticaria. - - - - - - 403
Consultations through the Journal. - - - 404
Arsenic sold for Antimony.—Empoisoning. - - 405
Murrain of Cattle. - - - - - 407
Tennessee Lunatic Asylum. - - - - 408
Electricity in Neuralgic Diseases. - - - 409
Death of Dr. Satterwhite. - - - - 409
Medical Institute of Louisville. - - - - 410

